# Advances in Mexico in the middle of the Decade of Action for Road Safety 2011–2020

**DOI:** 10.11606/S1518-8787.2018052000225

**Published:** 2018-07-17

**Authors:** Martha Híjar, Ricardo Pérez-Núñez, Aarón Salinas-Rodríguez

**Affiliations:** I Secretaría de Salud. Secretariado Técnico del Consejo Nacional para la Prevención de Accidentes. Ciudad de México, México; II Instituto Nacional de Salud Pública. Centro de Investigación en Evaluación y Encuestas. Cuernavaca, Morelos, México

**Keywords:** Motor Vehicles, Accidents, Traffic, prevention & control, Accidents, Traffic, trends, Accident Prevention, standards, Safety, legislation & jurisprudence, Accidentes de Tránsito, mortalidad y tendencias. Prevención de Accidentes, decenio de acción, México

## Abstract

**OBJECTIVE:**

To analyze the progress towards the accomplishment of the expected goal in the middle of the Decade of Action for Road Safety 2011–2020 in Mexico and its states.

**METHODS:**

This is a secondary analysis of road traffic deaths in Mexico between 1999 and 2015. We projected the trend for the period 2011–2020 using a time series analysis (autoregressive integrated moving average models). We used the value of the Aikaike Information Criterion to determine the best model for the national level and its 32 states.

**RESULTS:**

Mexico is progressing, approaching the proposed goal, which translates into 10,856 potentially prevented deaths in the five-year period from 2011 to 2015. This was due to a decrease in the number of deaths of motor vehicle occupants, as the deaths of pedestrians and motorcyclists were higher than expected. At least one third of the states had values below their goal; although the mortality rate remains unacceptably high in five of them. We identified four states with more deaths than those originally projected and other states with an increasing trend; thus, both cases need to strengthen their prevention actions.

**CONCLUSIONS:**

The analysis can allow us to see the progress of the country in the middle of the Decade of Action, as well as identify the challenges in the prevention of traffic injuries in vulnerable users. It contributes with elements that provide a basis for a need to rethink both the national goal and the goal of the different states.

## RESUMEN

**OBJETIVO:**

Analizar el avance de la meta esperada a mitad del Decenio de Acción para la Seguridad Vial 2011–2020 en México y sus entidades federativas.

**MÉTODOS:**

Análisis secundario de las muertes por accidentes de tránsito en México para el 1999–2015. Se proyectó la tendencia para el periodo 2011–2020 utilizando análisis de series de tiempo (modelos autorregresivos integrados de medias móviles). Se utilizó el valor del Criterio de Información de Aikaike para determinar el mejor modelo para el nivel nacional y sus 32 entidades federativas.

**RESULTADOS:**

México va avanzando cercano a la meta propuesta, lo que se ha traducido en 10,856 defunciones potencialmente prevenidas en el quinquenio 2011 a 2015. Esto ha sido a expensas de una disminución en el número de muertes de ocupantes de vehículos de motor; ya que las muertes en peatones y motociclistas han ido por arriba de lo que se esperaba. Al menos una tercera parte de las entidades federativas tuvo el número de defunciones por debajo de su meta; aunque en cinco de ellas la tasa de mortalidad continúa inaceptablemente alta. Se identificaron cuatro entidades con más muertes que las proyectadas originalmente y otras con tendencia al incremento donde se requiere, para ambos casos, fortalecer las acciones de prevención.

**CONCLUSIONES:**

El análisis realizado permite observar los avances del país a mitad del Decenio de Acción, así como identificar los retos en materia de prevención de lesiones causadas por el tránsito en usuarios vulnerables. Aporta elementos para soportar la necesidad de replantear tanto la meta nacional como la de las distintas entidades federativas.

## INTRODUCTION

The United Nations (UN), with Resolution 64/255, has proclaimed the period 2011–2020 as the Decade of Action for Road Safety. It also encouraged countries to join this global initiative to address the significant burden of road traffic injuries (RTI) in the world^1^. This initiative seeks to translate the elements highlighted in the 2004 World Report compiled by the WHO into public policies^2^. Mexico has been working on it since 2003, and since 2008 it has started working with the WHO/PAHO and other players within the framework of the Mexican Road Safety Initiative^3^. The country published, through the Ministry of Communications and Transport and the Ministry of Health, the Agreement by which it announced the National Road Safety Strategy 2011–2020^4^, based on the decade of action for road safety promoted by the WHO. As a result, the country has committed itself to reducing by 50% the number of deaths by RTI projected for 2020, as well as reducing as much as possible the injuries and disabilities associated with this public health problem^4^.

In the middle of the decade, two important events marked 2015 in the area of road safety. The first one was the establishment of a new development agenda for the next 15 years, approved at the seventh United Nations meeting in September, which gave rise to the so-called Sustainable Development Goals. As part of this agenda, two goals were set: goal 3.6 which establishes “[f]or 2020, the halving of the number of deaths and injuries caused by [traffic] in the world”, and goal 11.2 which establishes “[f]or 2030, the provision of access to safe, accessible, and sustainable transportation systems for all and improvement of road safety, in particular by expanding public transport, paying attention to the needs of vulnerable persons, women, children, persons with disabilities, and older adults”^5^. The second fact was the Brasilia Declaration, during the “Second High-Level Global Conference on Road Safety: Time for Results” carried out in November in the Brazilian capital^6^. In the inaugural address, Mexico became part of this Declaration, ratifying the commitments established by the country in the Decade of Action for Road Safety and the National Road Safety Strategy^a^. This presupposes the need to support this commitment with the analysis of progress and the outstanding aspects of the road safety agenda of the country.

The Ministry of Health, through the Technical Secretariat of the National Council for Accident Prevention, estimated in 2013 the number of deaths expected for the period 2012–2020. The “Trend” function of Excel^®^ was used, as well as data on traffic deaths observed from 2000 to 2011^7^. There was little discussion in the country about whether this approach was the most appropriate, particularly considering that the data for 2011 was included in the estimate, when in fact this year was already the beginning of this strategic period for road safety. This function uses an Ordinary Least Squares model that is not necessarily the best approach to take into account the self-correlation of the time series data. It was also not discussed at the national or global level what the most appropriate technical approach could be to identify the best projection for the number of deaths by RTI, and thus the elements to better evaluate progress in the context of the Decade of Action, which at the time of writing this article, is in its seventh year. The method chosen has important implications in terms of monitoring and evaluation of actions, as an inadequate estimate could overestimate or underestimate the gains achieved by the different countries.

Given the UN resolution, Mexico assumed, without any discussion, a commitment to reduce mortality by 50% in 10 years, also expecting that each state would establish its goal following this same logic. This was done without considering the potential impact of the actions being implemented or planned to be implemented during the Decade of Action and without realistically taking into account the availability of (human, material, and financial) resources in the country and in each of its states. These are key elements that should be considered when setting specific program goals after a thoughtful and serious analysis of the evidence on which road safety measures offer the greatest short-term preventive potential for the epidemiological profile of the country in a complex scenario of scarcity of available resources (cost-benefit analysis)^8^ ^,^ ^9^.

In an effort to motivate the academic discussion around this subject, this work aimed to analyze the progress of the expected goal in the middle of the Decade of Action for Road Safety 2011–2020 in Mexico and its 32 States. This information is fundamental to monitor and to evaluate the progress towards meeting the ambitious goal we set ourselves as a country.

## METHODS

This study has an observational design based on official statistics on deaths from road traffic injuries in Mexico in the period 1999–2015. The Decade of Action was determined for the decade 2011–2020, thus we used the information from 1999 to 2010 to project mortality by 2020. We used information from 2011 to 2015, the most recent data at the time of this analysis, to document the progress between the values programmed for those years and what is observed, at the country level and for the different states.

We used the mortality databases already validated by the National Institute of Statistics and Geography of Mexico, available on its website (http://www.beta.inegi.org.mx/proyectos/registros/vitales/mortalidad/). We extracted the number of recorded deaths per month at the national level and for each state. We took into account the place where the death occurred, which might not be the same as where the traffic event took place. Although the mortality database is considered by the WHO to be very good^10^, there are problems regarding the classification of deaths in unspecified codes that suggest the possibility that the real magnitude of the RTI issue may be underestimated^11^ ^,^ ^12^. In a minimal percentage of cases (0.07% of the cases in the period), the month of death was not recorded; this percentage was highest in 1999 (0.47%).

We used the official estimates of the population from the middle of the year of the National Population Council to calculate mortality rates (http://www.gob.mx/conapo).

The analysis of information on mortality trends for RTI was partially determined by two unique characteristics. The first one was the structure of information. As we have information about the number of deaths (counts) during fixed periods (continuous variable), we could model it according to incidence rates (mortality rates) using this double information; or we could explicitly model the observed series of deaths over time (or their corresponding rate) by the statistical techniques associated with the time series. The second one is the purpose of the analysis. The main objective of this study was to estimate the mortality for RTI in a period of 10 years (from information observed between 1999 and 2010). Thus, more emphasis was placed on prediction than on the model specification itself.

We carried out a Time Series Analysis. We constructed the monthly mortality rate per 100,000 inhabitants observed for the time series between 1999 and 2010, without including the observations without month of death. This information was modeled with interrupted time series using Autoregressive Integrated Moving Average (ARIMA) models, so that we could predict the trend of mortality rates from 2011 to 2020. These models take into account the correlation of the series of the monthly observations and the possible seasonality of the phenomenon observed. We analyzed the data separately for each of the 32 states of Mexico and aggregates at the national level.

We selected the best prediction model in two stages. We used some exploratory graphical instruments (correlograms), as well as the estimation of autocorrelation and partial autocorrelation (with a maximum specification of 12 lags) to determine both the autoregressive component and the moving average. Then, using the Aikaike Information Criterion (AIC), we identified the ARIMA model that best described the data^13^. This criterion indicates the model that has the lowest value in its value as the best model^14^. Thus, we selected the models that best reproduced the observed time series (evaluated graphically), and which, therefore, had a more accurate prediction of future values. In the end, these estimates were compared with the first estimate for the country^7^.

We evaluated the progress during the Decade of Action and estimated the number of deaths potentially avoided, taking as reference the trend estimated by the ARIMA models and the data observed for the period 2011–2015. This exercise allowed us to evaluate the magnitude and implications of the differences observed by the two methods used. We evaluated the observed trend in mortality for the national level by type of road user and compared it to the projection performed.

We used the statistical package Stata 14^®^ for all analyses.

## RESULTS

**Figure 1:**
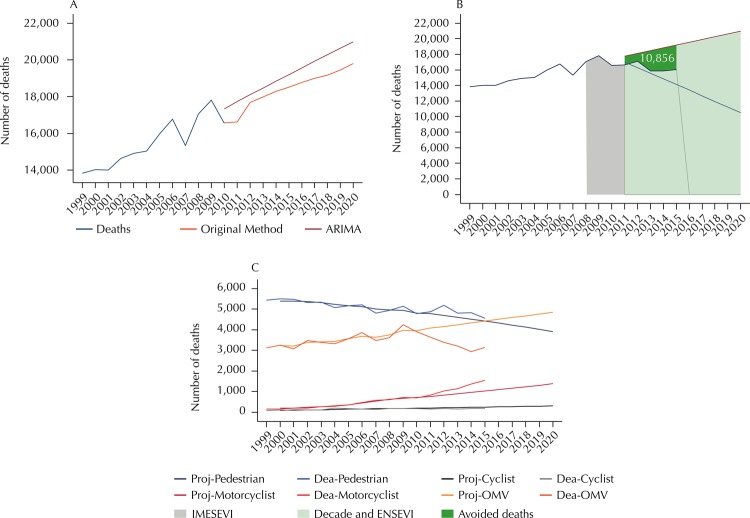
Projection of road traffic deaths for 2020 and progress in the fulfillment of the goal of the Decade of Action for Road Safety. Mexico, 2015.

ARIMA: Autoregressive integrated moving average; Proj: projection; Dea: deaths; OMV: occupants of motor vehicles with 4 or more wheels; IMESEVI: Mexican Road Safety Initiative; Decade: Decade of Action for Road Safety 2011–2020; ENSEVI: National Road Safety Strategy 2011–2020 in Mexico

The Table shows the projection of the number of deaths from RTI in Mexico using ARIMA models. At the national level, the ARIMA model projected 20,984 deaths by 2020, which is higher than the value from the original method (19,810) (Figure 1, A). Mexico has moved closer to the goal established for the Decade of Action, which supposes the possibility of having prevented 10,856 deaths in 2011–2015 (Figure 1, B). This gain was due to a significant reduction in the number of deaths of motor vehicle occupants, which was lower than the value estimated using the same methodology as the aggregate data. However, the number of deaths of motorcyclists since 2011 was higher than expected, taking into account the trend observed between 1999 and 2010. This was similar to the deaths observed in pedestrians, which, although in smaller magnitude, were above the projected value (Figure 1, C).

**Table:** Descriptive analysis of Mexico and its States.

State	Total population		Projection of the number of deaths from traffic injuries for 2020					Observed deaths^a^		Prevented deaths^b^	
	2010	2020	Original method	ARIMA model				2010	2015	Original method	ARIMA
				Forecasting	Specification	AIC	BIC				
National	114,255,559	127,091,642	19,810	20,984	AR(1) S(12) MA(1 12)	-249	-234	16,559	16,039	7,555	10,856
Aguascalientes	1,195,788	1,369,306	267	364	AR(1) S(12) MA(1 12)	214	229	216	200	145	347
Baja California	3,224,844	3,729,225	335	139	AR(1 2) S(12) MA(1 2 12)	63	83	271	464	0	0
Baja California Sur	649,617	878,830	171	204	AR(1) S(12) MA(1 3 4 12)	362	383	130	131	142	165
Campeche	836,748	974,877	101	138	AR(1) S(12) MA(1 12)	246	260	96	136	0	34
Coahuila	2,782,013	3,129,782	399	349	AR(5) S(12) MA(5 12)	107	122	371	312	0	0
Colima	658,910	782,831	161	164	AR(9) S(12) MA(9 12)	234	248	112	132	55	46
Chiapas	4,903,754	5,568,648	563	267	AR(1 2 12) S(12) MA(1 12)	7	27	171	668	0	0
Chihuahua	3,525,273	3,882,739	897	980	AR(1 12) S(12) MA(1 2 12)	157	177	801	506	881	1,049
Mexico City	8,944,599	8,738,914	656	707	AR(1) S(12) MA(1 12)	-73	-59	1,026	768	96	227
Durango	1,669,814	1,847,547	544	539	AR(10 11) S(12) MA(10 11 12)	174	194	319	388	243	83
Guanajuato	5,558,502	6,033,559	1,125	1,238	AR(1 11 12) S(12) MA(1 11 12)	82	105	1,012	935	508	823
Guerrero	3,444,265	3,657,305	481	410	AR(1) S(12) MA(1 12)	62	76	372	458	0	0
Hidalgo	2,690,086	3,044,937	575	589	AR(3) S(12) MA(3 12)	114	128	348	395	283	232
Jalisco	7,442,625	8,363,277	1,678	1,787	AR(9 12) S(12) MA(9 12)	-17	0	1,516	1,236	1,037	1,471
State of Mexico	15,571,680	18,075,065	2,048	2,104	AR(4 5 7) S(12) MA(5 12)	-198	-178	1,784	1,572	848	1,021
Michoacán	4,420,270	4,741,317	971	1,229	AR(1 3 12) S(12) MA(1 3 12)	99	122	865	497	1,363	2,000
Morelos	1,803,340	2,030,580	198	253	AR(1 12) S(12) MA(1 12)	159	177	203	238	0	111
Nayarit	1,108,861	1,333,853	348	496	AR(2) S(12) MA(9 12)	315	330	301	213	309	638
Nuevo Leon	4,723,272	5,440,278	594	763	AR(1 2 11 12) S(12) MA(1 2 3 12)	31	60	325	640	0	0
Oaxaca	3,868,108	4,127,899	661	802	AR() S(12) MA(10 12)	71	83	595	543	508	873
Puebla	5,863,823	6,481,536	733	820	AR(11) S(12) MA(11 12)	13	28	734	788	96	280
Querétaro	1,848,191	2,147,765	457	584	AR(4) S(12) MA(4)	243	255	416	327	291	586
Quintana Roo	1,350,945	1,798,603	161	69	AR(1 10) S(12) MA(1 10 12)	206	226	130	176	7	0
San Luis Potosí	2,616,459	2,868,906	428	640	AR(1) S(12) MA(1 3 7 12)	139	159	445	450	0	342
Sinaloa	2,851,334	3,105,704	1,060	852	AR(1 2 5) S(12) MA(1 2 5 12)	130	156	684	688	557	85
Sonora	2,727,032	3,125,865	818	990	AR(3) S(12) MA(1 12)	153	168	672	480	768	1,173
Tabasco	2,252,641	2,498,558	782	940	AR(4 12) S(12) MA(4 12)	87	105	576	654	112	501
Tamaulipas	3,334,664	3,735,589	442	533	AR(1 12) S(12) MA(1 11)	178	196	546	613	0	131
Tlaxcala	1,186,143	1,363,576	236	199	AR(3) S(12) MA(1)	292	304	182	200	56	0
Veracruz	7,712,247	8,328,389	1,002	1,109	AR(1 2 12) S(12) MA(1 2 3 12)	-51	-25	650	576	820	1,235
Yucatán	1,980,691	2,252,505	364	482	AR(3 5) S(12) MA(5 12)	120	137	287	281	211	450
Zacatecas	1,509,020	1,633,878	355	393	AR(5) S(12) MA(5 12)	252	267	403	374	0	32

ARIMA: Autoregressive integrated moving average; AIC: Akaike information criterion; BIC: Bayesian Information Criterion; AR: autoregressive component; S: seasonality; MA: moving average component

^a^ It includes observations without month of death.

^b^ It corresponds to the number of potentially avoided deaths between 2011 and 2015.

We identified states where there was no progress in terms of avoided deaths from RTI, taking into account the ARIMA predictions. On the other hand, Michoacan, Jalisco, Veracruz, Sonora, Chihuahua, and the State of Mexico seem to be the states with the highest health progress (Table).

**Figure 2:**
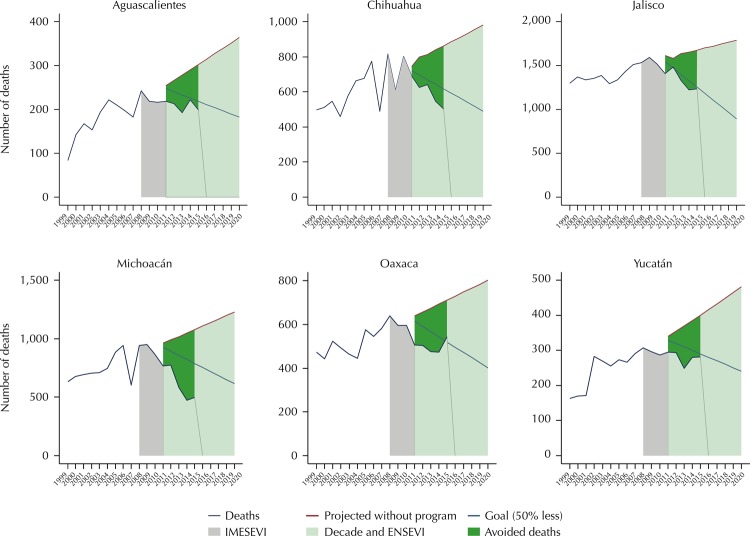
Mexican states that adequately progress in the fulfillment of the goal of the Decade of Action for Road Safety 2011–2020.

IMESEVI: Mexican Road Safety Initiative; Decade: Decade of Action for Road Safety 2011–2020; ENSEVI: National Road Safety Strategy 2011–2020 in Mexico

Six states had a good performance in the number of deaths observed in the context of the National Road Safety Strategy 2011–2020 (Figure 2). Three of them showed a clear decreasing trend between 2011 and 2015, below the established goal: Chihuahua, Jalisco, and Michoacán. In the other three, we observed a stabilization in the number of deaths. The data for Jalisco and Michoacán in 2014 were the lowest for the whole period, although we observed a slight increase in both cases for 2015.

**Figure 3:**
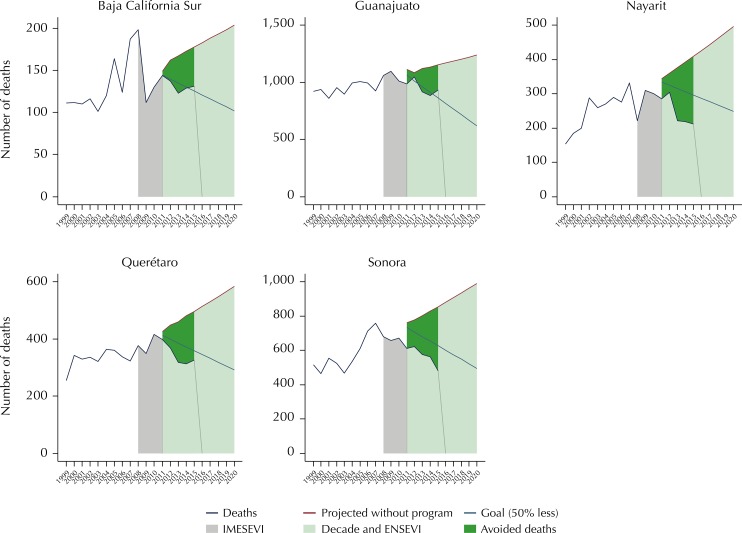
Mexican states that adequately progress in the fulfillment of the goal of the Decade of Action for Road Safety 2011–2020 but which still have high mortality rates.

IMESEVI: Mexican Road Safety Initiative; Decade: Decade of Action for Road Safety 2011–2020; ENSEVI: National Road Safety Strategy 2011–2020 in Mexico

**Figure 4:**
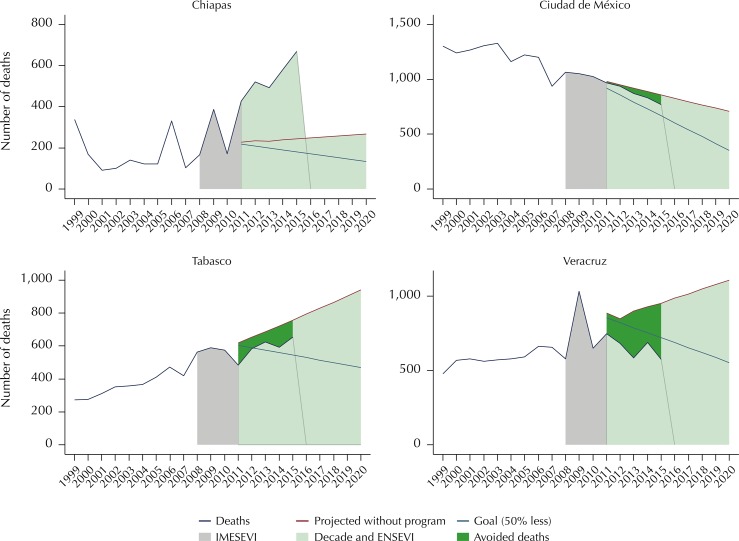
Atypical progress in different states. Mexico, 1999–2015.

IMESEVI: Mexican Road Safety Initiative; Decade: Decade of Action for Road Safety 2011–2020; ENSEVI: National Road Safety Strategy 2011–2020 in Mexico

Other states, although performing well in relation to their goal, continued to have a high mortality rate for RTI for 2015, above 16 per 100,000 inhabitants. This was the case for Baja California Sur (17.1), Guanajuato (16.1), Nayarit (17.4), Querétaro (16.3), and Sonora (16.4) (Figure 3). On the other hand, we observed atypical advances. The state of Chiapas seems to have fallen far from its goal and even from the mortality projection carried out by the ARIMA model, which already showed an increasing trend. Mexico City was far from meeting its goal; however it showed, after Veracruz (7.2), the lowest mortality rate for 2015, with 8.7 per 100,000 inhabitants. Tabasco is a serious case for the country. It showed an increasing trend between 2011 and 2015 and it was the state with the highest mortality rate in the country for several years, as well as for 2015, with 27.4 per 100,000 inhabitants. Veracruz showed an apparent good performance being below the goal of the Decade of Action (Figure 4).

**Figure 5:**
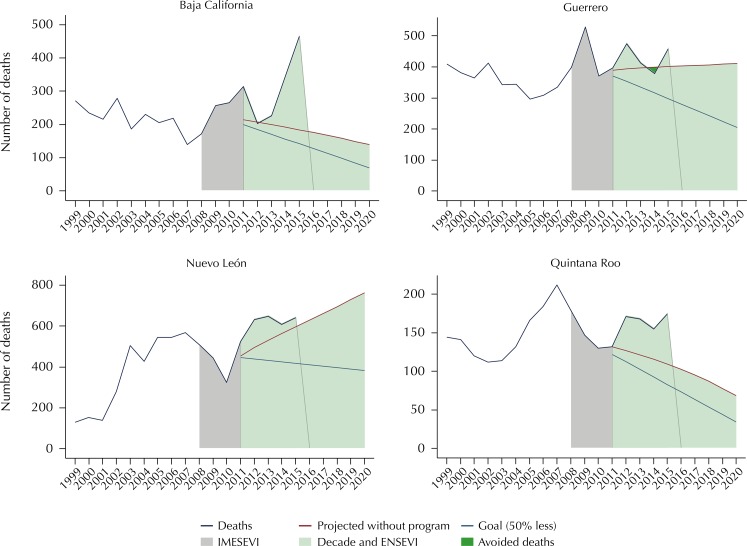
Road safety regress in different States. Mexico, 1999–2015.

IMESEVI: Mexican Road Safety Initiative; Decade: Decade of Action for Road Safety 2011–2020; ENSEVI: National Road Safety Strategy 2011–2020 in Mexico

Four states apparently regressed in the subject of road safety (Figure 5). Guerrero, Nuevo León, and Quintana Roo went above the projection, despite showing a mortality rate below the national rate in 2015: 12.8, 12.6, and 11.2, respectively. Baja California presented a worrying increase in its mortality rate between 2013 and 2015, and the value for 2015 was the highest in the whole period.

## DISCUSSION

This work is an important medium-term analysis exercise for the possibility of reaching the goal of the Decade of Action for Road Safety by 2020. From the time series analysis, we could calculate that Mexico may have prevented 10,856 deaths during the first five years. These gains seem to be attributable to national efforts that have tended to prioritize preventive actions for motor vehicle occupants, which showed a greater decrease. It is necessary, however, to evaluate to what extent changes in the pattern of mobility also explain these changes. While stabilization in the number of deaths is a major step forward, there is a clear need to move forward by decreasing the high number of persons who still die each year from RTI in the country.

It is important to use an appropriate method when setting goals for health programs, especially when using country-wide measures and specific projections for state. For this end, it is necessary to take into account the structure of the data analyzed as well as the purpose of the analysis (modeling, forecasting, etc.). The first estimate could be underestimating the trend observed for the period 1999–2010, showing a potentially underestimated projection and a goal that, for practical purposes, is even more ambitious. From this analysis, the method originally used was not the best approach. We believe that our estimate could also be an underestimate for two reasons. The first one is due to actions linked to the Mexican Road Safety Initiative which have been set since 2008 in Mexico, before the Decade of Action, which could have affected the trend that was observed in the previous period and influenced the establishment of national and state goals. The second one is due to the decrease observed in 2007, which, as documented^11^ ^,^ ^12^, could be more related to problems in the recording of information that affected this year than to a real decrease in mortality.

The goal of reducing by 50% the number of deaths associated with RTI in the country, which was the WHO proposal for all countries, makes sense if we do not to lose sight of the fact that it is an ideal to be achieved (although quite ambitious for the installed capacity and resources for preventive actions). The establishment of this same goal for all states without considering their epidemiological profile, the mortality trend in the previous period, the preventive potential of the different interventions implemented locally, and the different resources available as recommended^8^ ^,^ ^9^, is not the most appropriate path. The country and the different states should set realistic goals based on this diagnosis.

The setting of the same goal precludes recognizing the progress made before the Decade of Action for Road Safety. Mexico City has promoted different road safety actions since well before the Decade, which could explain the sustained decrease in its mortality rate in the analyzed period^15^. Among the actions promoted in advance, we can mention the adaptations to its traffic legislation to meet the different risk factors^16^ ^,^ ^17^, particularly on drink-driving with police sobriety checkpoints carried out as part of the “Drive Without Alcohol” program^18^, regulation of pre-hospital medical care with the Regulatory Center for Medical Emergencies^19^, and policies to promote mass public transport^20^, among others. The use of the estimated trend for Mexico City (n = 707, rate of 8.09 per 100,000 inhabitants) would be a great gain or an appropriate goal to be achieved as it is lower than expected for the country itself (n = 10,492 or rate of 8.25 per 100,000 inhabitants). However, intending to decrease the estimated number of deaths by 50% by 2020 (n = 353) would mean that this state should have a mortality rate similar to what is currently observed, for example, in Sweden, which, we believe, would be unrealistic.

After accepting the WHO recommendation to reduce by 50% the number of deaths from RTI, another approach to establish the national goal could be setting the rate at 8.25 per 100,000 inhabitants as the goal for each state (equivalent to the national goal). This would make it possible to highlight which states face greater challenges in terms of road safety. Examples are the states whose increasing trend is very noticeable (Aguascalientes, Colima, Chiapas, Durango, Guerrero, Sinaloa, Tabasco, Tlaxcala) and which should go even lower than the originally set goal. This is because mortality rates associated with this goal remain higher than desirable.

There are other states that could fall victim to their own gains. The clearest case is Jalisco, where the reduction observed in 2010 could be the result of the work carried out in the context of the Mexican Road Safety Initiative^3^ ^,^ ^21^ ^,^ ^22^ supported by resources from the Bloomberg Global Road Safety Program since 2008 and more clearly from 2010, the year with a first decrease^23^, even though previous work have not shown statistically significant effects in the short term^24^ ^,^ ^25^. These decreases, which could be the result of these efforts, influence the estimated trend, setting a lower goal than what would have been established if the trend observed between 2004 and 2009 was kept by the lack of actions.

Much of the initiatives promoted by the Mexican Road Safety Initiative and the early years of the Decade focused mainly on motor vehicle occupants. Significant decreases could be observed when compared to the expected trend without the different actions of road safety being promoted. In this sense, the data presented show the need to not continue using aggregate rates and also work on improving road safety, with interventions focused on vulnerable users such as pedestrians, cyclists, and especially motorcyclists. For these road users, the observed data are higher than expected before the Decade of Action.

Beginning in 2015, concrete actions have been taken to promote road safety among vulnerable users in the country, such as an intervention guide for the prevention of injuries in urban cyclists^26^, the promotion of the implementation of road safety audits for vulnerable users^27^, and the promotion of a intervention model to promote road safety for motorcyclists^28^ including the development and publication of an Official Mexican Standard Project that establishes minimum standards for motorcycle helmets^29^. In addition, different cities on their own initiative have promoted programs to promote the use of active transportation (ECOBICI, MIBICI, among other programs)^30^ or to improve road safety for pedestrians, such as the Mexico City Safe Pass program^31^. The effects of these actions can be verified in the final part of the Decade of Action for Road Safety.

The data presented here do not recall previous exercises on the underestimation of road traffic mortality, thus the results presented here should be taken with caution^11^ ^,^ ^12^. For example, the results presented in this paper show that Veracruz is fulfilling its established goal, when in fact the actual value could be higher than observed given the large number of deaths allocated to unspecified codes or “garbage codes”. Baja California and particularly Chiapas should be carefully analyzed. They should be evaluated in relation to what extent the increase observed between 2010 and 2014 responds more to improvements in the death record and not to a real increase in the health damages associated with this public health problem. The gains observed in Michoacán could be related to a slight increase in the underestimation of mortality in recent years.

Despite the limitations mentioned above, this analysis contributes to the national and international literature by documenting the case of Mexico regarding the progress made in the middle of the Decade of Action for Road Safety 2011–2020. This evidence can be used as an input to push forward the work in road safety and discuss the need or not to rethink the goal of the health sector program related to reducing the mortality rate for RTI and eventually achieve the ambitious goal that we propose as a country in the National Road Safety Strategy 2011–2020^4^.

## Footnotes

a Speech by Mrs. Ambassador of Mexico to Brazil, Beatriz Paredes, in the inauguration of the Second High-Level Conference on Road Safety: Time for Results. November 18, 2015.
